# Dynamics of temporal immune responses in nonhuman primates and humans immunized with COVID-19 vaccines

**DOI:** 10.1371/journal.pone.0287377

**Published:** 2023-10-19

**Authors:** Resmi Ravindran, Harsharonjit Kang, Cindy McReynolds, Gursharan Kaur Sanghar, W. L. William Chang, Santhamani Ramasamy, Afsal Kolloli, Ranjeet Kumar, Selvakumar Subbian, Bruce D. Hammock, Dennis J. Hartigan-O’Connor, Aamer Ikram, Angela Haczku, Imran H. Khan

**Affiliations:** 1 Department of Pathology and Laboratory Medicine, University of California, Davis, Davis, California, United States of America; 2 Department of Entomology and Nematology, University of California, Davis, Davis, California, United States of America; 3 Pulmonary, Critical Care and Sleep Medicine, University of California, Davis, Davis, California, United States of America; 4 California National Primate Research Center, University of California, Davis, Davis, California, United States of America; 5 Public Health Research Institute, New Jersey Medical School, Rutgers University, Newark, New Jersey, United States of America; 6 National Institutes of Health, Islamabad, Pakistan; University of Virginia, UNITED STATES

## Abstract

We assessed the humoral immune responses to a COVID-19 vaccine in a well-controlled rhesus macaque model compared to humans immunized with two mRNA vaccines over several months post-second dose. The plasma IgG levels against seven coronaviruses (including SARS-CoV-2) and antibody subtypes (IgG 1–4 and IgM) against SARS-CoV-2 were evaluated using multiplex assays. The neutralization capacity of plasma antibodies against the original SAR-CoV-2 isolate and nine variants was evaluated in vaccinated humans and non-human primates. Immunization of macaques and humans with SARS-CoV-2 vaccines induced a robust neutralizing antibody response. In non-SIV-infected adult macaques immunized with an adenoviral vector expressing S-RBD (n = 7) or N protein (n = 3), elevated levels of IgG and neutralizing antibodies were detected 2 weeks post-second dose. Immune responses to the S-RBD vaccine in SIV-infected adult macaques (n = 2) were similar to the non-SIV-infected animals. Adult humans immunized with Pfizer (n = 35) or Moderna (n = 18) vaccines developed IgG and neutralizing antibodies at 4 weeks post-second dose. In both vaccine groups, IgG 1 was the predominant subtype, followed by IgG 3. The IgG levels, including total and IgG 1,2,3 elicited by the Moderna vaccine, were significantly higher than the corresponding levels elicited by the Pfizer vaccine at 4 weeks post-second dose. A significant correlation was observed between the plasma total IgG antibody levels and neutralization titers in both macaques and humans. Furthermore, broad-spectrum neutralization antibodies against several variants of SARS-CoV-2 were detected in the plasma of both macaques and humans after two vaccinations.

## Introduction

Severe acute respiratory syndrome coronavirus 2 (SARS-CoV‐2) was first identified in Wuhan, China, in December 2019 and is responsible for the present COVID-19 pandemic. Until the beginning of May 2023, there were about 766 million confirmed cases of COVID-19, including 6.9 million deaths; about 13.3 billion vaccine doses have been administered worldwide [1]. Exposure to SARS-CoV-2 can cause different clinical outcomes ranging from asymptomatic infection to mild-to-moderate, and severe disease manifestations, such as acute respiratory distress syndrome (ARDS), vascular and neurological complications, and eventually death [1–3]. The severity of the worldwide impact of the COVID-19 pandemic on humans calls for rapid actions, principally oriented toward a global vaccination campaign and the development of effective intervention strategies.

The US-Food and Drug Administration (FDA), on August 23, 2021, gave the first approval of a messenger RNA (mRNA) vaccine BNT162b2 (Pfizer-BioNTech now marketed as Comirnaty) to protect against the progression of SARS-CoV-2 infection and on January 31, 2022, approved a second vaccine, mRNA-1273, developed by Moderna [2, 3]. These vaccines are administered as three shots, intramuscularly, given at 3 and 24 weeks apart for Pfizer-BioNTech and at 4 and 24 weeks apart for Moderna. They have been shown to offer protection by triggering an immune response against the SARS-CoV-2 spike (S) protein [2–6]. Since these vaccines deliver mRNA encoding only for SARS-CoV-2 S protein, the expected elicited response is the production of anti-S immunoglobulin G (IgG), IgM, and IgG subclasses, particularly against the receptor-binding domain (RBD) of the S protein which contains many neutralizing epitopes [7–9]. These vaccines have shown variable efficacy against SARS-CoV-2 variants [10–16].

Assessment of the immunogenicity elicited by the vaccines in a large cohort of vaccinated individuals is an essential priority for the scientific community to understand the correlates of protection and to improve the protective efficacy. More than ninety-one million cases of COVID-19 have been documented in the USA, and high seropositivity rates have been observed in recent studies [17]. Although the immune response against SARS-CoV-2 has been documented in humans with natural infection [18–21], the development of immunity after the administration of mRNA vaccines is not completely understood. For instance, there is only limited data available on simultaneous analysis and comparison of antibodies against SARS-CoV-2 (S-RBD, and nucleocapsid (N) proteins), and S proteins of SARS-CoV, MERS, and the four common coronaviruses (229E, NL63, OC43, and HKU1) in healthy volunteers receiving two mRNA vaccines. The induction of cross-reactive antibodies to seasonal β-coronaviruses such as OC43 and HKU1 by SARS-CoV-2 mRNA vaccination has been reported [22]. In the present study, we have simultaneously measured antibodies against S or RBD proteins of SARS-CoV-2, SARS-CoV, MERS-CoV, and four common human coronavirus strains, and nucleocapsid (N) protein of SARS-CoV viruses in individuals who received the mRNA vaccines. In addition to total IgG (SARS-CoV-2 S-RBD), we have measured IgM, and IgG subtypes- 1, 2, 3, and 4 in the study subjects.

In addition, the comparative analysis of COVID-19 vaccine-induced immune response between animal models and humans has yet to be deciphered. Non-human primate (NHP) virus-challenge models are critical to understanding the pathogenesis and host immune response conferred by viral infections, such as SARS-CoV-2, which are not easily addressed or feasible in humans. Furthermore, the NHP models have proven as a valuable tool for assessing the immunogenicity and protective efficacy of COVID-19 vaccines [23–25]. In the present study, we used the NHP model to evaluate the immunogenicity of an adenoviral vector-based vaccine expressing S-RBD protein.

Several studies have reported an increased risk of death and severity of COVID-19 in people living with HIV [26–28]. Even though near-complete immune recovery is expected in people living with HIV who are under antiretroviral therapy (ART) [28], defective B-cell functioning, not completely recovered by ART, might contribute to decreased COVID-19-vaccine response. At present, a low number of studies exist on antibody responses and their duration in people living with HIV. In the present study, we have evaluated the humoral immune response to S-RBD adenoviral vector vaccine in SIV-infected adult macaques to determine whether cART treatment impacts the vaccine-induced immune responses.

Currently, the US-FDA does not recommend SARS-CoV-2 antibody tests to assess the level of protection provided by an immune response to COVID-19 vaccination [29]. However, to understand the efficacy of COVID-19 vaccines, we must use appropriately validated assays to characterize the antiviral immunity associated with the vaccines. Thus, longitudinal post-vaccination studies of healthy volunteers are critical for establishing the “levels of protection” offered by a vaccine candidate. Previous studies have reported the elicitation of high levels of serum anti-SARS-CoV-2 IgG and neutralizing antibody responses post-second dose of mRNA vaccines and the progressive decrease in antibodies following vaccination [30]. However, the long-term immunity and decreased antibody levels associated with breakthrough SARS-CoV-2 infections after vaccination was not fully characterized.

The World Health Organization (WHO) has classified SARS-CoV-2 variants based on their transmissibility and/or pathogenicity as variants of concern (Alpha (B.1.1.7), Beta (B.1.351), Gamma (B.1.1.248), and Delta (B.1.617.2) and variants of interest (Epsilon (B.1.427/B.1.429), and Kappa (B.1.617.1) [31]. Alpha, Beta, Epsilon, Kappa, and Gamma variants contain D614G mutation [32], while the Beta and Delta plus variants have K417N in the RBD [32, 33]. The vaccine resistance to Alpha, Beta, and Gamma variants was attributed to the N501Y and E484K mutations [32, 34]. The notorious Omicron variant shares some of the mutations (K417N, N501Y, and D614G) with other SARS-CoV-2 variants like Alpha, Beta, Kappa, Delta, and Gamma [32]. We assessed the neutralization capacity against SARS-CoV-2 wild type and nine variants in vaccinated humans and non-human primates.

We previously reported multiplex microbead immunoassays-based antibody detection against SARS-CoV-2, SARS-CoV, MERS-CoV, and common human coronavirus strains (229E, NL63, OC43, HKU1) and have demonstrated its potential for sensitive, specific, and efficient serodiagnosis in natural SARS-CoV-2 infection [35]. The current study provides a detailed characterization of humoral responses elicited in the plasma of healthy human volunteers immunized with Pfizer or Moderna vaccines and assesses the kinetics of antibody-mediated immunity against SARS-CoV-2 wild type and its variants following vaccination. We also sought to evaluate the humoral response to the adenoviral vector-based COVID-19 vaccine platform in SIV and non-SIV-infected adult macaques and qualitatively compared it to the vaccine-mediated immune response in human adults.

## Materials and methods

### Ethics statement

All methods were carried out in accordance with relevant guidelines and regulations reviewed and approved by the relevant institutions.

Human plasma samples from COVID-19 patients, vaccinated and healthy individuals were obtained under the protocols approved through the relevant Institutional Review Boards (IRBs) at the University of California, Davis Medical Center, National Institute of Health (NIH), Pakistan as well as School of Biological Sciences (SBS), University of the Punjab, Lahore, Pakistan (IRB 1584225, IRB 218204, IRB 1617547–1, IRB 1758309–1). Written informed consent was received from all participants before inclusion in the study, and all the samples were de-identified before access.

Non-human primates (adults) were housed according to the Guide for the Care and Use of Laboratory Animals and the standards outlined by the American Association for Accreditation of Laboratory Animal Care; all animal experiments were performed under approval from Institutional Animal Care and Use Committees (IACUC) at the UCDavis [35, 36]. NHPs were maintained in cages with 4 square feet of floor space, or 6 square feet if over 10 kg, with fixed perch bars in a temperature-controlled vivarium with continuous monitoring of temperature and humidity. All animals had visual and auditory access to other macaques 24 hours per day and were fed a balanced commercial macaque chow (Purina Mills, Gray Summit, MO) twice daily with fresh produce twice weekly, and free access to water 24 hours per day. Supplemental food was provided when clinically indicated. Environmental enrichment was provided daily, including manipulanda (forage boards, mirrors, puzzle feeders) and novel foodstuffs. Veterinarians, animal health technicians, and staff technicians conducted daily health/clinical assessments of animals. For vaccinations and blood collections, animals were anesthetized by intramuscular injection (i.m) of ketamine-HCl (Parke-Davis, Morris Plains, NJ) at 10 mg/kg of body weight. For virus inoculation and nasal secretion sample collection, animals were additionally anesthetized with 15–30 ug/kg dexmedetomidine HCl injected i.m. and anesthesia was reversed with 0.07–0.15 mg/kg atipamezole HCl injected i.m. Analgesics were given to minimize pain and discomfort at the discretion of the veterinary staff and nutritional supplements were administered, as necessary. When euthanasia was necessary, animals were humanely euthanized at the end of the study by a barbiturate overdose, and necropsy procedures were performed by veterinary pathologists and support staff. Criteria for assessments of the health and well-being of the animals were as follows: no signs of injury, distress, or pain that cannot be alleviated by analgesics, weight loss, hypothermia, persistent anemia, chronic dehydration, lethargy, severe dyspnea, neurological deficits, coagulopathies, motor retardation, etc. If an animal’s physical condition deteriorated prior to the scheduled endpoint, clinical veterinary staff would have euthanized the animal following the Guidelines for Humane Euthanasia of Animals on Projects (GHEAP) at the CNPRC. No animals were infected with SIV for purposes of these studies but were previously infected for pathogenesis or vaccine work before treatment with antiretroviral therapy. In this study, none of the macaques were euthanized for welfare reasons before completion. The study was not terminal.

### Rhesus macaque (*Macaca mulatta*) model

Plasma samples were obtained from macaques immunized with adenoviral vector vaccine expressing SARS-CoV-2 S-RBD protein (n = 7) collected at baseline, 2 and 4 weeks post-first and -second dose. To model the humoral immune response to COVID-19 vaccination in HIV infection, two SIV-infected macaques under combination antiretroviral therapy (cART) were included in the study. These macaques also received an adenoviral vector vaccine expressing SARS-CoV-2 S-RBD, and plasma samples were collected at baseline, 2 and 4 weeks post-first dose, and 2, 4, and 6 weeks post-second dose. They did not show any AIDS symptoms, and their CD4 counts were normal with undetectable plasma SIV RNA.

In addition, longitudinal plasma samples were obtained from 3 rhesus macaques immunized with adenoviral vector vaccine expressing SARS-CoV-2 nucleocapsid protein (n = 3) at baseline, 2 weeks post-first dose, and 2, 4, 6, and 8 weeks post-second dose. These macaques lacked antibodies to SARS-CoV-2 S-RBD and N at week 0 (baseline).

Samples from 116 healthy, seronegative macaques were used in the study. Ninety-five were archived plasma samples collected before 2019 and cryopreserved at -80C [37]. The first group consists of rhesus macaques (n = 58) obtained from a specific pathogen‐free (SPF) colony at the CNPRC at UC Davis. Plasma samples from the second group consisted of healthy rhesus macaques (n = 37) at the AAALAC‐accredited SPF colony at the Charles River Laboratories (Wilmington, MA). In addition, post-pandemic plasma samples from twenty-one healthy rhesus macaques from CNPRC were included.

### Human subjects

#### COVID-19 patients and controls

Plasma samples from RT-PCR-confirmed COVID-19 patients from the University of California, Davis, were obtained as previously described [35]. As negative controls, we used plasma samples from healthy individuals (n = 26) collected before the COVID-19 pandemic as described previously [35, 38, 39] and post-pandemic (n = 52).

#### Plasma from individuals immunized with Pfizer and Moderna vaccines

SARS-CoV-2 vaccinations started in the USA at the end of December 2020 with Pfizer-BioNTech BNT162b2 mRNA and Moderna mRNA-1273 vaccine. We conducted a longitudinal study in which participants (ages 21 to 60 years) were recruited among the University of California, Davis staff, before receiving two doses of the Pfizer or Moderna vaccine. The plasma samples were collected from EDTA-treated blood, before or on the day of the first vaccine dose, 4 weeks post-first vaccine dose, and 4 and 26 weeks post-second vaccine dose (n = 18 (Moderna); 3 weeks post-first vaccine dose, and 4 and 26 weeks post-second vaccine dose (n = 35 (Pfizer). Twenty-eight of these healthy subjects participated in the Pfizer vaccine trial in August 2020 in the Sacramento region. All the participants were without a history of SARS-CoV-2 infection at the time of sample collection. Two participants in the Pfizer group became positive for SARS-CoV-2 by RT-PCR three weeks post-second dose vaccination.

### Microbead coating with human coronavirus (HCoV) antigens

Recombinant viral antigens for microbead coating were obtained from BEI Resources or Sino Biological Inc. (Wayne, PA). The following antigens were produced under Federal contract HHSN272201400008C and obtained through Biodefense and Emerging Infections Research (BEI) Resources Repository, NIAID, NIH: SARS-CoV-2 Spike (S) glycoprotein receptor-binding domain (S-RBD) (NR-52366); SARS-CoV S Protein (NR-722); SARS-CoV-2 Nucleocapsid (N) protein (NR-53246) and SARS-CoV N protein (NR-48761). MERS-CoV S-RBD (40071-V08B1). 229E S Protein (40601-V08H); NL63 S Protein (40600-V08H); HKU1 S Protein (40021-V08H); and OC43 S Protein (40607-V08B) were obtained from Sino Biological Inc. (Wayne, PA) as previously described [35]. Carboxylated microbeads were purchased from Luminex Corp. (Austin, TX). Various antigen preparations were covalently conjugated to the microbeads as previously described [35].

### Multiplex microbead immunoassay

Multiplex assays were performed based on the xMAP platform (Luminex Corp, Austin, TX), and data (median fluorescence intensity (MFI) were collected as previously described [35]. One modification in assay performance was made where all sera and biotinylated-secondary antibodies were diluted in 1% casein (Vector Laboratories, Burlingame, CA) instead of prionex. Briefly, sera/plasma samples were diluted 1:200 in 1% casein and incubated with HCoV antigen-coated beads for 1 hr. at room temperature in a 96-well plate. After incubation, the beads were washed twice by adding 100 μl of wash buffer (PBS-tween) per well and drained under vacuum using a vacuum manifold (Millipore Corporation, Bedford, MA). To detect human and macaque IgG, phycoerythrin-conjugated anti-human IgG was used (Jackson Immuno Research, Pennsylvania) at a 1:500 dilution in PBS-tween and incubated at room temperature for 15 min. Following incubation, beads were washed two times with wash buffer, resuspended in 100 μl of wash buffer per well, and analyzed in the Magpix instrument.

To detect IgM and the IgG subtypes, the multiplex assay was performed using only SARS-CoV-2 S-RBD and N, SARS-CoV S & N proteins. For the detection of human IgM, biotinylated anti-human IgM was used (BD Biosciences Cat# 555781) at a 1:500 dilution, and for the detection of antigen-specific antibodies per IgG subclasses, biotinylated IgG1 (Southern Biotech Cat# 9052–08) was added at 5ug/ml, and biotinylated IgG2, IgG3, or IgG4 were added, along with specific detection reagents (Southern Biotech Cat#s 9052–08, 9060–08, 9210–08, 9200–08) at 20 ug/ml in 1% casein and the assay was performed as described previously [35].

### Bio-Plex Pro bead-based SARS-CoV-2 neutralization antibody assay (surrogate neutralization assay (sVNT))

The Bio-Plex Pro Human SARS-CoV-2 Variant Neutralization Antibody 11-Plex Panel (Bio-Rad^™^ Laboratories, #12016897) was used to assess vaccine-induced antibody response against two SARS-CoV-2 wildtype strains and an early clinical strain YP_009724390.1, isolated from Wuhan, China (S1 and RBD), and 9 variants (Alpha S1, Beta S1, Gamma RBD, Kappa RBD, Epsilon RBD, D614G S1, N501Y RBD, E484K RBD, and K417N RBD). sVNT allows the indirect detection of SARS-CoV-2 neutralizing antibodies through the determination of antibody blocking of S-RBD-ACE2 interaction. This type of surrogate assay has been demonstrated to have a good correlation with levels of neutralizing antibodies determined by whole virus or pseudovirus neutralization assays [9, 40]. The assay was performed according to the manufacturer’s instructions (Bio-Rad^™^ Laboratories, Hercules, CA).

#### Bio-Plex sVNT assay optimization of human samples

The accuracy of the Bio-Plex sVNT was evaluated to see how it performed under our conditions. Plasma samples from four COVID-19 patients collected over several days post-symptoms were selected to compare sVNT and WVNT. For the sVNT assay, plasma samples were serially diluted to determine the optimal dilution that gave the highest correlation between % inhibition of the neutralizing antibodies with WVNT. Serial dilutions of various plasma samples were done as follows: 1:5, 1:25, 1:37.5, 1:56.3, 1:84.4, and 1:125 (see S1 Fig). Samples were incubated with SARS-CoV-2 antigen-coupled beads that compete with biotinylated ACE-2 receptors to bind neutralizing antibodies in plasma samples in a competitive assay. The percentage inhibition was calculated from the MFI values of the samples versus that of the negative control (a well that had coupled beads, sample diluent, bio-ACE2 receptor, and streptavidin-phycoerythrin) using the formula: Percentage inhibition = 1 –(MFI of the sample / MFI of the negative control) x 100.

Optimal sample dilution was selected to ensure other similar samples would be predicted to fall within the range and not report as saturated percent inhibition. A sample dilution of 1:37.5 was chosen as the optimal dilution for sVNT assays (S2 Fig). Subsequent analysis of the study samples was performed at 1:37.5 dilutions. sVNT was used to evaluate vaccine efficacy in the samples from individuals immunized with Pfizer and Moderna vaccines. WVNT titers and sVNT (% inhibition) were highly correlated in human COVID-19 patients: RIB00016 (R^2^ = 0.99), RIB00004 (R^2^ = 0.94), RIB00020 (R^2^ = 0.86) and RIB00012 (R^2^ = 0.78) (S2 Fig).

#### Macaque samples

sVNT was used to evaluate vaccine efficacy in adult macaques (S3 Fig).

### Data analysis

For the analysis of antibody data, cut-off values were calculated for each antigen-coated microbead set using data from healthy individuals (Cut-off = Mean MFI + (3 standard deviations)). Separate cut-off values were determined for each secondary reagent (IgG, IgM, IgG1,2,3,4). The cut-off values were used to determine antibody-positive samples in the data sets. For measurements of antibodies and to compare differences between multiple groups, data were assessed with 1-way ANOVA and Tukey’s multiple comparison test using GraphPad Prism software (Prism, CA, USA). Graphs were generated, p-values were determined by using the GraphPad Prism software, and adjusted p-values were reported. For all assays, p<0.05 between groups was considered statistically significant.

## Results

### Multiplex antibody profiling in a rhesus macaque model

The multiplex antibody assay was employed to analyze humoral immune responses (the panel included seven human coronaviruses, as described in Methods) in the plasma of non-SIV and SIV-infected animals immunized with SARS-CoV-2 S-RBD protein.

#### Total IgG antibodies against SARS-CoV-2 S-RBD

**Fig 1:**
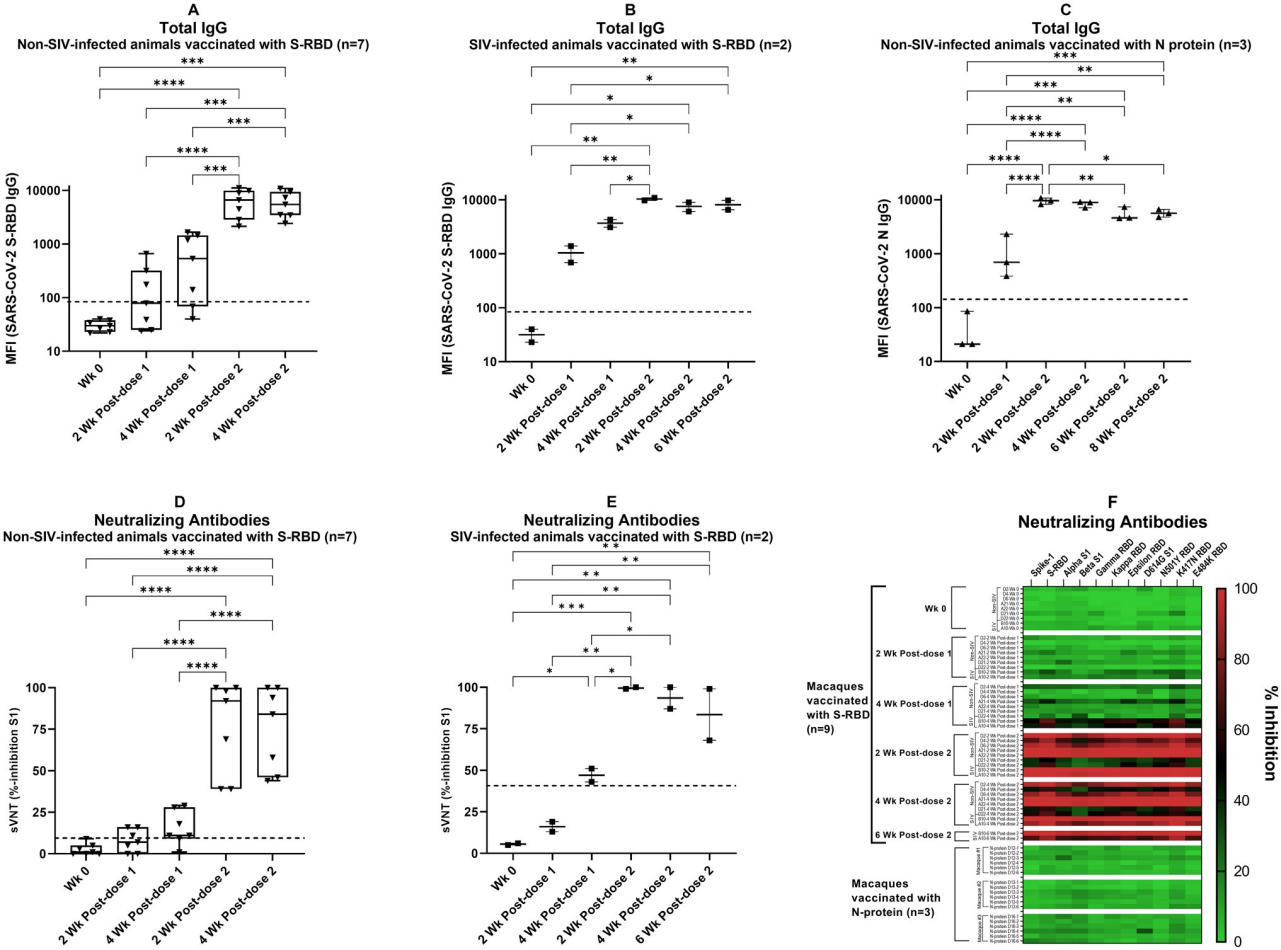
Antibody responses against SARS-CoV-2 S-RBD or N protein in macaques immunized with adenoviral vector vaccines. (A) Non-SIV infected (n = 7), and (B) SIV-infected (n = 2), immunized with S-RBD protein. (C) Non-SIV infected immunized with N protein (n = 3). Multiplex antibody assay was performed to detect antibodies against the following antigens: SARS-CoV-2 S-RBD & N, SARS-CoV S & N, MERS-CoV S-RBD, and S proteins of four common coronaviruses. Only antibodies against SARS-CoV-2 proteins are shown; antibodies against other antigens in the panel were undetectable except SARS-CoV S, N, and OC43 (see S1 Table in S1 File). MFI values (log10) with median and interquartile range (IQR) are shown (Fig 1A-1C). Dashed lines indicate the cut-off values of the assay for SARS-CoV-2 S-RBD (Fig 1A & 1B) or N (Fig 1C) IgG. Neutralizing antibodies against SARS-CoV-2 S1 elicited by S-RBD vaccine in (D) non-SIV infected animals, (E) SIV-infected animals. % inhibition for SARS-CoV-2 S1 obtained using the sVNT assay with median and IQR are shown (Fig 1D & 1E). Dashed lines indicate the cut-off values of the sVNT assay. p values by one-way ANOVA and Tukey’s multiple comparison test between the groups are as follows: *p<0.05, ** p<0.01, *** p<0.001, **** p<0.0001. (F) Heat map of the neutralization capacity (% inhibition) of macaque sera against SARS-CoV-2 wildtype (S1 and S-RBD) and nine variants of either S1 or RBD protein subunits. The arrangement of samples is the same as indicated in Fig 1A-1C. Each row in the heatmap corresponds to one sample and columns correspond to SARS-CoV-2 wild type or variants in the Bio-Plex sVNT assay. The color intensity scale represents the % inhibition values ranging from the highest (100; red) to no % inhibition (0; green).

In non-SIV-infected animals (n = 7), anti-SARS-CoV-2 S-RBD antibodies (IgG) were detectable at 2 weeks post-first dose in three macaques (Median MFI = 79; IQR:32, 248) (Fig 1A). At four weeks post-first dose, five macaques had anti-SARS-CoV-2 S-RBD antibodies (Median MFI = 535; IQR:105, 1322). Strong antibody response to SARS-CoV-2 S-RBD was detectable in all macaques at 2 weeks (Median MFI = 6625; IQR:3674, 9477) and 4 weeks (Median MFI = 5489; IQR:3488, 8368) post-second dose immunization (Fig 1A).

We also studied antibody responses in SIV-infected macaques (n = 2) under combination antiretroviral therapy (cART) vaccinated with an adenoviral viral vector expressing SARS-CoV-2 S-RBD protein (Fig 1B). Antibodies against S-RBD were detectable at 2 weeks post-first dose (Median MFI = 1042; IQR:866, 1217) and steadily increased at 2 weeks post-second dose immunization (Median MFI = 10356; IQR:10063, 10650). However, the antibody responses were stable at 4 weeks (Median MFI = 7571; IQR:6842, 8299) and 8 weeks (Median MFI = 8157; IQR:7348, 8965) post-second dose immunization (Fig 1B). Since the number of animals in the SIV group (n = 2) was less compared to the non-SIV group (n = 7), the variations in antibody levels and neutralization titer should be interpreted cautiously.

#### Total IgG antibodies against SARS-CoV-2 N protein

In non-SIV infected rhesus macaques immunized with adenoviral vector vaccine expressing N protein (n = 3), SARS-CoV-2 N specific antibodies (IgG) were detectable at 2 weeks post-first dose immunization in all three macaques (Median MFI = 693; IQR:539, 1494) (Fig 1C). SARS-CoV-2 N-specific antibodies peaked at 2 weeks post-second dose immunization (Median MFI = 9636; IQR:8929, 10212). Even though antibody levels dropped by week 6 post-second dose (Median MFI = 5623; IQR:5198, 6118), the levels were significantly higher (p-value = 0.0042) compared to 2 weeks post-first dose immunization (Fig 1C). Plasma from most healthy controls (n = 116) did not contain antibodies to S-RBD or N proteins (S2 Table in S1 File); in two macaques the SARS-CoV-2 S-RBD and N antibodies were slightly above the baseline cut-off value.

#### Neutralizing antibodies against S1 or S-RBD display a broad-spectrum effect against several strains

sVNT assay was performed (described in Methods) to evaluate the neutralizing antibody titers (% inhibition against SARS-CoV-2 wild type and nine variants) in animals immunized with S-RBD. Neutralizing antibodies were detected in two animals in the non-SIV and SIV-infected group at 2 weeks post-first dose, which further increased 4 weeks post-first dose vaccination (Fig 1D and 1E). Titers for three animals in the non-SIV infected group remained low compared to other animals in this group at all tested time points post-vaccination (Fig 1D). These animals also had the lowest total IgG at all time points post-vaccination. However, a high neutralizing antibody titer was observed at 2 weeks that persisted at 4 weeks post-second dose in both SIV and non-SIV groups (Fig 1D and 1E). Neutralization capacity against the 9 SARS-CoV-2 variants was similar to the wildtype S1, and S-RBD in macaques immunized with adenoviral vector vaccine expressing S-RBD (Fig 1F). Neutralization capacity against the beta variant was the lowest among the tested SARS-CoV-2 variants (Fig 1F). Non-SIV infected rhesus macaques immunized with adenoviral vector vaccine expressing N protein did not exhibit neutralization antibody titers against SARS-CoV-2 wild type or any of the tested variants.

Neutralizing antibody titer and total IgG in animals immunized with S-RBD (R^2^) were strongly correlated with R^2^ = 0.93 for the non-SIV-infected group and R^2^ = 0.96 for the SIV-infected group (S3A and S3B Fig, respectively).

### Multiplex antibody profiling in humans immunized with Pfizer and Moderna mRNA vaccines

#### Total IgG antibodies against SARS-CoV-2 S-RBD

**Fig 2:**
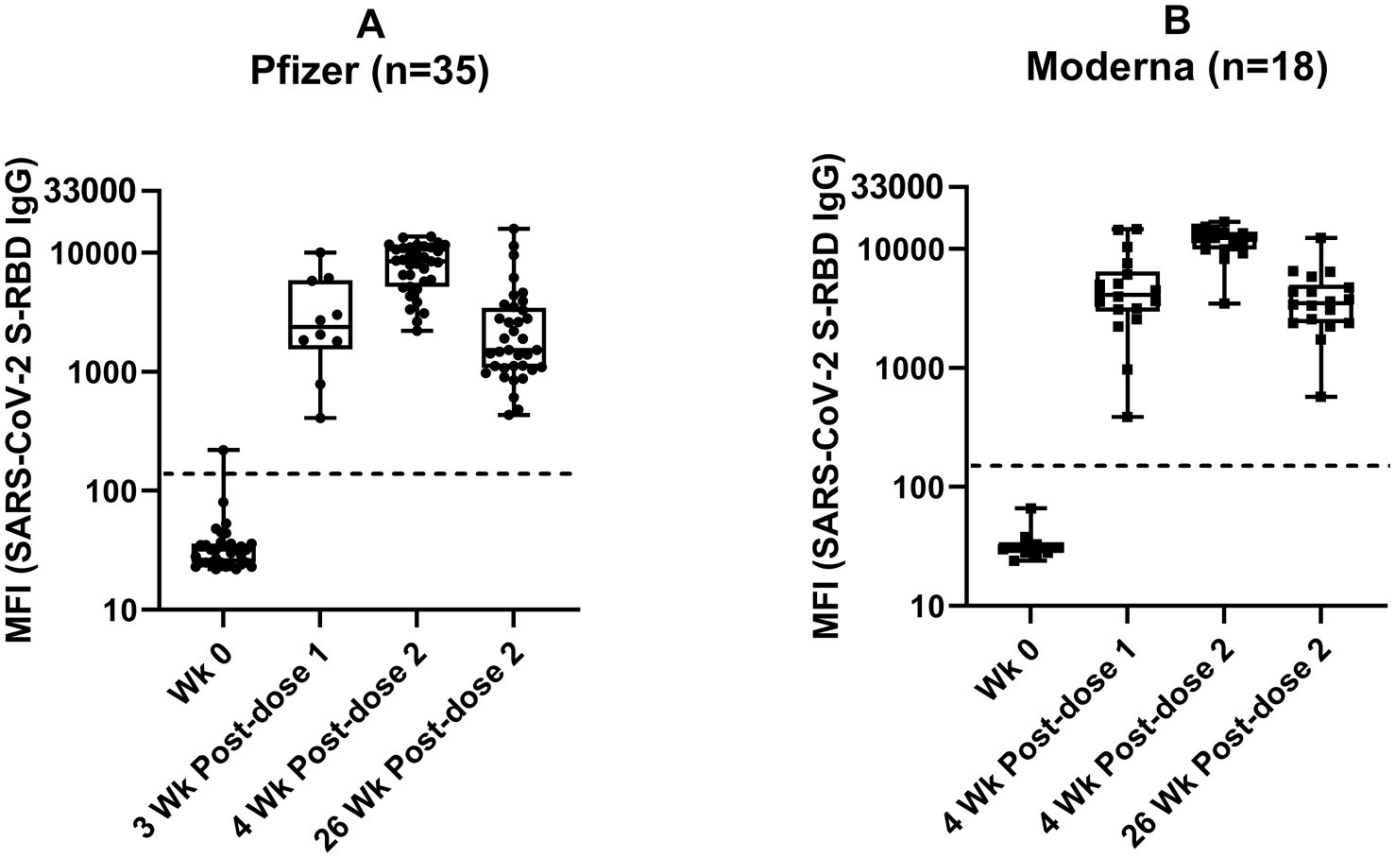
Antibodies (IgG) against SARS-CoV-2 S-RBD in humans immunized with mRNA vaccines. A) Pfizer, and B) Moderna. The description of the multiplex antibody assay is as in Fi 1. MFI values (log10) with median and IQR are shown. The samples taken at various intervals are as follows: Pfizer- week 0 (Baseline n = 33), 3 weeks post-first dose (n = 10), 4 weeks post-second dose (n = 35), and 26-weeks post-second dose (n = 36), and Moderna—week 0 (Baseline n = 10), 4 weeks post-first dose, 4 and 26-weeks post second-dose (n = 18). The dotted line indicates the assay cut-off values calculated using healthy controls (n = 101).

Multiplex assay was employed to measure the levels of IgG against SARS-CoV-2 S-RBD in healthy adult volunteers immunized with the Pfizer (n = 35) and Moderna (n = 18) mRNA vaccines (Fig 2A and 2B). Plasma samples were collected at baseline, 3 weeks post-first dose (Pfizer only), 4 weeks post-first dose (Moderna only), and 4 and 26 weeks post-second dose (both groups).

At baseline, none of the participants had antibodies against S-RBD except for one individual in the Pfizer group. This individual had S-RBD antibodies slightly above the cut-off value but devoid of SARS-CoV-2 N antibodies throughout the study (Described in detail in the section SARS-CoV-2 N (IgG) under Results).

Antibodies against S-RBD were detectable in all the volunteers three weeks post-first dose in the Pfizer (Median MFI = 2376; IQR:1838, 5795) (Fig 2A) and four weeks post-first dose in the Moderna group (Median MFI = 4110; IQR:3113, 5852) (Fig 2B). Antibodies peaked at 4 weeks post-second dose (for Pfizer group Median MFI = 8465; IQR:3278, 11016 and Moderna group Median MFI = 12392; IQR:10029, 13609) and maintained at lower levels at 26 weeks post-second dose vaccination in both groups (for Pfizer group Median MFI = 1526; IQR:1089, 3342 and Moderna group Median MFI = 2504; IQR:2424, 4636), as reported previously [41]. Interestingly, antibody titers were waning in both groups at 26 weeks post-second dose (Fig 2A and 2B). Although antibody levels dropped, these individuals remained antibody-positive throughout the study, and levels observed were similar to what was seen post-first dose, and the drop in MFI was not statistically significant compared to post-first dose levels (p-value = 0.9299 (Pfizer), p-value = 0.5242 (Moderna).

The levels of antibodies in the Moderna group were significantly higher than in the Pfizer group only at 4 weeks post-second dose (p-value 0.0004), not at 3–4 weeks post-first dose or 26 weeks post-second dose (p-values = 0.7125 and 0.8823, respectively).

#### Total IgG antibodies against SARS-CoV-2 N

Multiplex assay was employed to measure the levels of antibodies against N protein. At baseline (week 0), none of the participants had antibodies against N protein in both groups except one individual in the Moderna group. This individual had antibodies against N protein slightly above the cut-off, while the S-RBD IgG level was below the baseline (S4B Fig). The same individual remained positive for N and S-RBD IgG during post-first and second dose vaccination. None of the other participants had antibodies above the cut-off level at the post-first or second dose in the Moderna group.

Among the Pfizer group, none of the participants had antibodies above the cut-off level at the post-first or second dose except for one participant who had antibodies against N and S-RBD at 4 weeks post-second dose and remained positive until 26 weeks post-second dose vaccination (S4A Fig). This volunteer might have been exposed to SARS-CoV-2 during the study period. In addition, two participants in the Pfizer group became positive for SARS-CoV-2 by RT-PCR three weeks post-second dose vaccination and contained antibodies to SARS-CoV-2 N protein until 28–32 weeks post-second dose (S4A Fig).

### Kinetics of seroconversion and role of antibody subtypes

**Fig 3:**
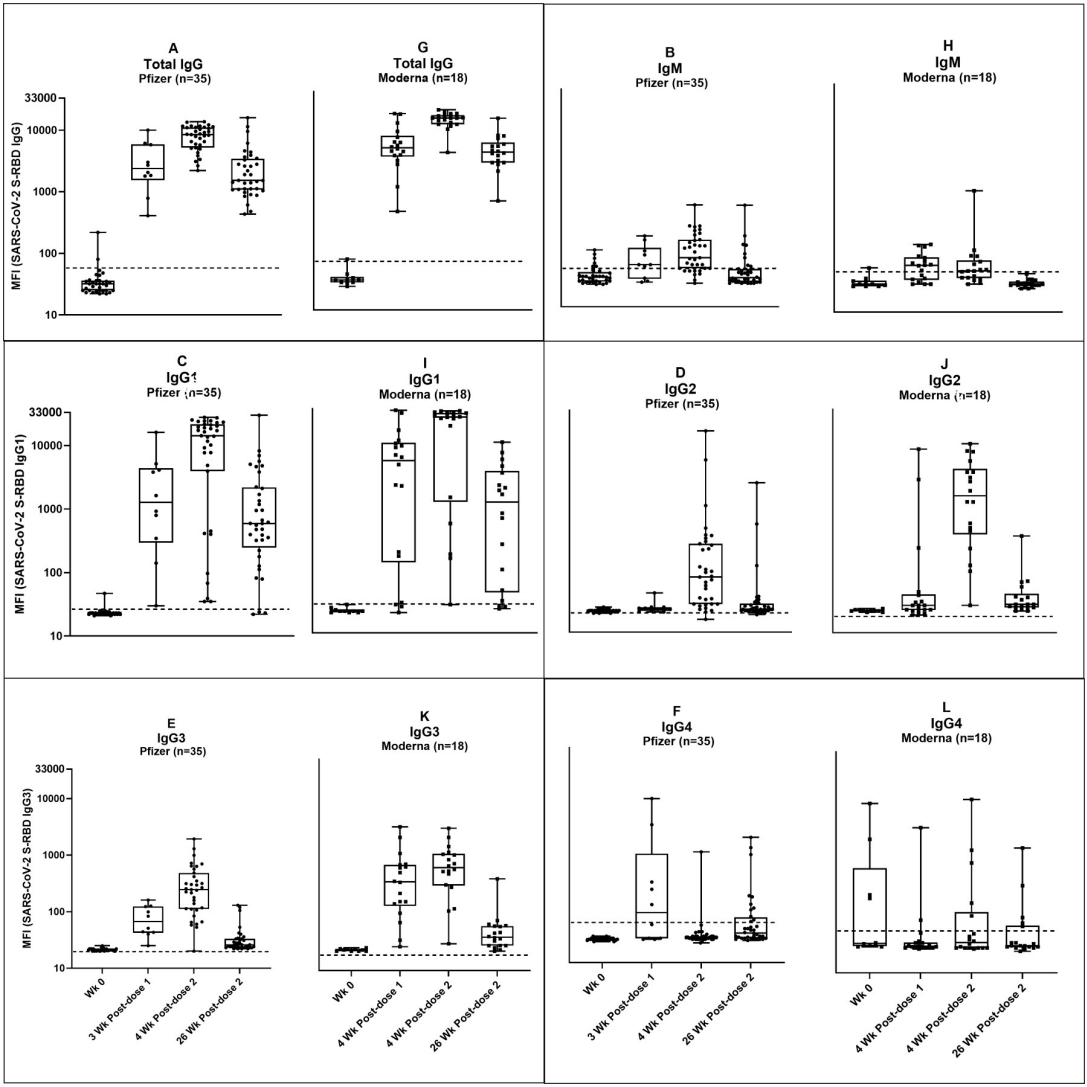
Kinetics of antibody profiles: Total IgG, IgM, and IgG subtypes-1, 2, 3, and 4 against SARS-CoV-2 S-RBD in individuals immunized with Pfizer (A-F) and Moderna (G-L) vaccines. MFI values (log10) with median and IQR are shown. Each antibody subtype was individually detected in the multiplex assays against the following antigens: SARS-CoV-2 S-RBD and N, SARS-CoV S & N proteins. Only antibodies against SARS-CoV-2 S-RBD are shown. The dotted line indicates the assay cut-off calculated using healthy controls (n = 43). The description of sample time points is as in Fig 2.

We also measured IgM, and IgG subtypes (IgG1, IgG2, IgG3, IgG4) responses to S-RBD in plasma samples from individuals immunized with Pfizer or Moderna vaccines (Fig 3A–3F (Pfizer); 3G-3L (Moderna)).

S3 Table in S1 File indicates the % of antibody positives and how many individuals developed detectable levels of plasma IgM, total IgG, and subtypes against S-RBD in Pfizer and Moderna groups at each time point post-vaccination. In general, a low level of IgM antibodies was detected in both groups (Fig 3B and 3H, and S3 & S4 Tables in S1 File). Sixteen individuals in the Pfizer group had low levels of IgM (Fig 3B) compared to one in the Moderna group (Fig 3H) at 4 weeks post-second dose immunization. Similarly, at 26 weeks post-second dose, four individuals in the Pfizer group (Fig 3B) had low levels of IgM compared to none in the Moderna group (Fig 3H). Rest of the individuals had IgM levels below the cut-off (Fig 3B and 3H).

In both vaccine groups, the total IgG (Fig 3A and 3G) response post-first and second dose was contributed by an elevation of IgG1 (Fig 3C and 3I), followed by IgG3 (Fig 3E and 3K). The IgG1 (Fig 3C and 3I) antibodies persisted until 26 weeks post-second dose vaccination, even though the levels dropped from the previous sampling time point. The IgG3 (Fig 3E and 3K) antibody levels were also dropped precipitously at 26 weeks post-second dose vaccination.

At 3 or 4 weeks post-first dose vaccination, 9 out of 10 individuals in the Pfizer group (Fig 3C and S3 Table in S1 File) and 14 out of 18 individuals in the Moderna group had elevated levels of IgG1 (Fig 3I and S3 Table in S1 File). At 4 weeks post-second dose, 32 out of 35 individuals in the Pfizer group (Median MFIs = 1277; IQR: 797, 4122) and 17 out of 18 individuals in the Moderna group (Median MFIs = 5049; IQR: 167, 9216) had elevated levels of IgG1. At 26 weeks post-second dose, 33 out of 35 individuals in the Pfizer group (Median MFIs = 592; IQR: 389, 1905) and 14 out of 18 individuals in the Moderna group (Median MFIs = 1125; IQR: 60, 2967) had elevated levels of IgG1. The median levels of IgG1 antibodies in all three post-vaccination time points in the Moderna group (Fig 3I) were as follows, Median MFIs = 5049 (IQR 167,9216), 24544 (IQR 5449, 28067), 1125 (IQR: 60, 2967). The median levels of IgG1 antibodies in all three post-vaccination time points in the Pfizer group (Fig 3C) were as follows, Median MFIs = 1277 (IQR 797, 4122), 14243 (IQR: 4410, 21308), 592 (IQR 389, 1905). The results were statistically significant only at 4 weeks post-second dose vaccination (p-value = <0.0001), and not at 3–4 weeks post-first dose or 26 weeks post-second dose (p-values = 0.384 and 0.8606, respectively).

A low level of IgG2 was observed in most of the Pfizer group (Fig 3D); although at 4 weeks post-second dose (Median MFI = 75; IQR:28, 247), three individuals had elevated levels of IgG2 (MFIs = 1018, 5423,15589). Two individuals in the Moderna group (Fig 3J) had elevated levels of IgG2 at four weeks post-first dose vaccination (MFI = 2585, 7786). The IgG2 levels in the Moderna group (Median MFI = 1718, IQR: 413, 3392) were significantly higher compared to the Pfizer group (Median MFI = 75, IQR: 28, 247) only at 4 weeks post-second dose (p-value = 0.0117), and not at 3–4 weeks post-first dose or 26 weeks post-second dose (p-values = 0.9697 and >0.9999, respectively).

Similarly, IgG3 levels in the Moderna group (Fig 3K) were significantly higher (Median MFI = 483, IQR: 302, 859) compared to the Pfizer group (Fig 3E) (Median MF = 246, IQR: 112, 450) at 4 weeks post-second dose (p-value = 0.0496). At 4 weeks post-first dose, the IgG3 levels were as follows: Pfizer group (Median MFI = 67, IQR: 44, 123) and Moderna group (Median MFI = 301, IQR: 132, 564) (p-value = 0.0514). At 26 weeks post-second dose, IgG3 levels dropped in both groups (p-value >0.9999). At this time point, 18 out of 35 individuals in the Pfizer group and 15 out of 18 individuals in the Moderna group had detectable levels of IgG3 (S3 and S4 Tables in S1 File).

Four individuals in the Pfizer group had detectible levels of IgG4 (Fig 3F) at three weeks post-first dose vaccination (MFIs = 208, 1808, 4842, 158) (S3 and S4 Tables in S1 File). In this group, one individual at 4 weeks post-second dose (MFI = 646) and 5 individuals at 26 weeks post-second dose vaccination had IgG4 antibodies (MFIs = 585, 760, 124, 119, 1122) (S3 and S4 Tables in S1 File). In the Moderna group (Fig 3L), four individuals had IgG4 antibodies at baseline (MFIs = 153, 5001, 175, 1341) (S3 and S4 Tables in S1 File). In this group, one individual at 4 weeks post-first dose (MFI = 2062), 4 individuals at 4 weeks (MFIs = 897, 129, 564, 5797), and 2 individuals at 26 weeks post-second dose (MFIs = 245, 975) vaccination had IgG4 antibodies (S3 and S4 Tables in S1 File).

### SARS-CoV-2 neutralization titers

**Fig 4:**
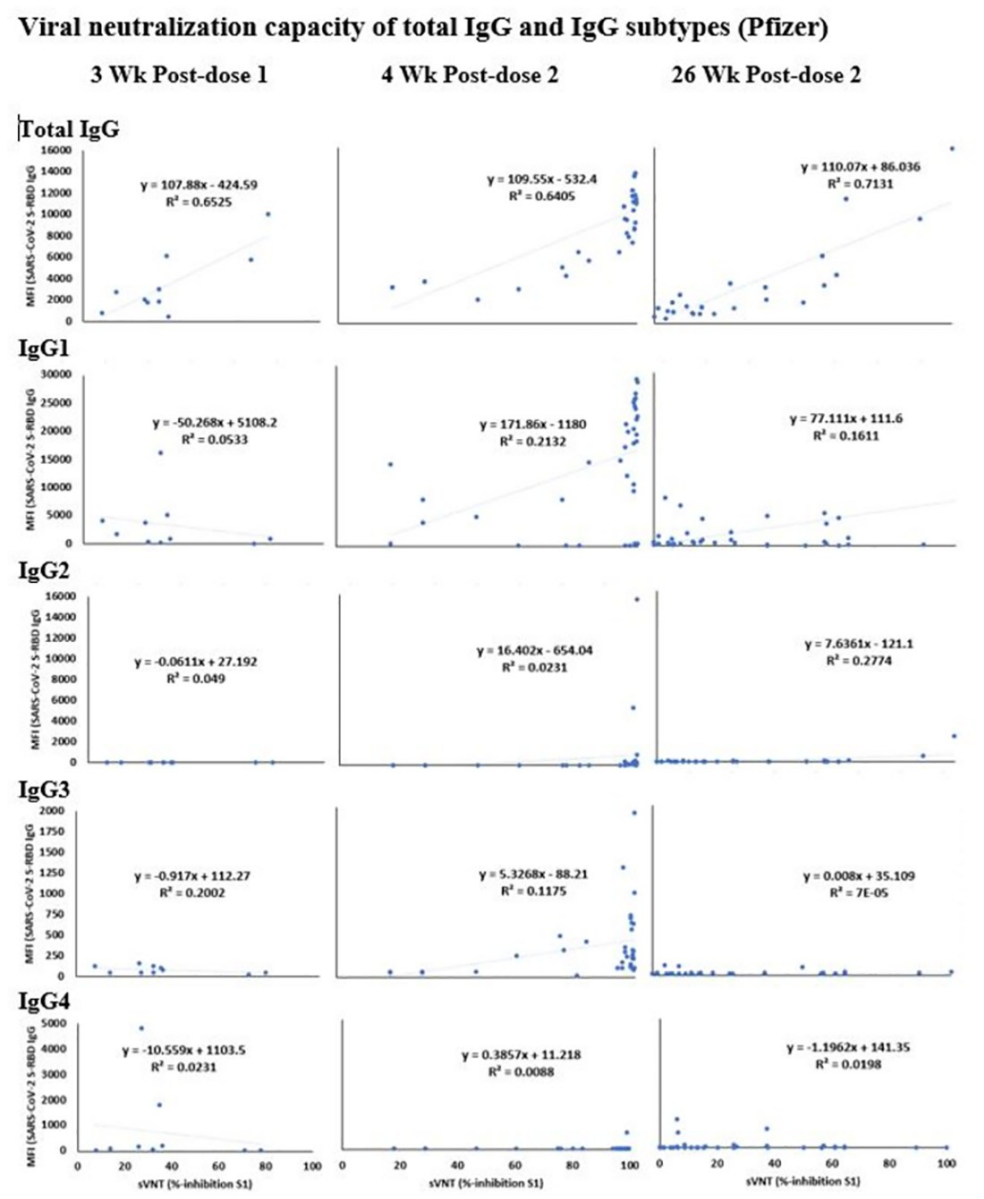
Correlation of neutralization capacity of antibodies against SARS-CoV-2 S1 (% inhibition) and IgG antibodies against SARS-CoV-2 S-RBD (MFI values) in the plasma samples, collected longitudinally from individuals immunized with Pfizer vaccine. The description of the multiplex antibody assay is as in Fig 3. The R^2^ values are shown as an indicator of the correlation between the neutralization potency and total IgG, IgG subtypes-1, 2, 3, and 4. The description of sample time points is as in Fig 2.

**Fig 5:**
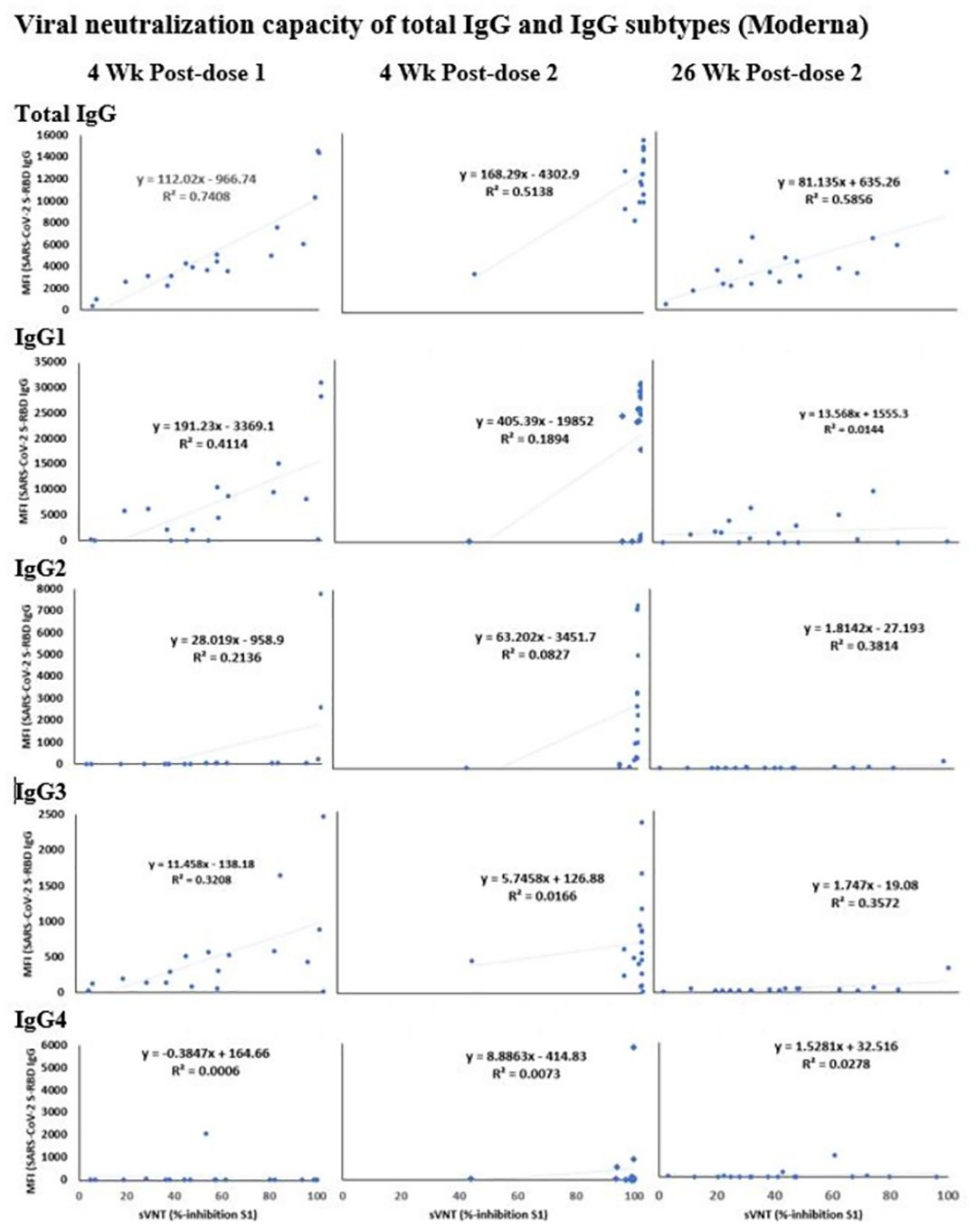
Correlation of neutralization capacity of antibodies against SARS-CoV-2 S1 (% inhibition) and IgG antibodies against SARS-CoV-2 S-RBD (MFI values) in the plasma samples, collected longitudinally from individuals immunized with Moderna vaccine. Measurements of IgG specific to SARS-CoV-2 S-RBD are plotted in the graphs. The description of the multiplex antibody assay is as in Fig 3. R^2^ values are shown as an indicator of the correlation between the neutralization potency and total IgG, and IgG subtypes-1, 2, 3, and 4. The description of sample time points is as in Fig 2.

The viral neutralizing antibodies were detected in individuals immunized with Pfizer and Moderna vaccines using a sVNT assay. The MFI values of S-RBD IgG were plotted against the % inhibition of neutralizing antibodies to SARS-CoV-2 S1 for the corresponding samples (Figs 4 and 5).

### Viral neutralization capacity of total IgG and IgG subtypes (Pfizer)

Interestingly, a strong-to-medium correlation was found between the neutralization capacity and IgG levels (Total) in the Pfizer (Fig 4) group in all time points post-vaccination (R^2^ = 0.65, 0.64, and 0.71). In addition, a low correlation was noted in the neutralization capacity between IgG 1, 2, and 3 at all tested time points post-vaccination (R^2^ values <0.28) (Fig 4).

Similar to the Pfizer group, a strong-to-medium correlation was found between neutralization capacity and IgG levels (Total) in the Moderna (Fig 5) groups at all three tested time points post-vaccination (R^2^ = 0.75, 0.51, and 0.59). At 4 weeks post-first dose, a modest correlation was noted between neutralization capacity and the levels of IgG1 and IgG3 (R^2^ = 0.4 and 0.3, respectively) in the Moderna group. The correlation was low for the other tested time points in all IgG subtypes in the Moderna group (R^2^ values <0.41).

### Viral neutralization capacity of total IgG and IgG subtypes (Moderna)

#### Neutralizing antibodies against S1 or S-RBD display a broad-spectrum effect against several strains

**Fig 6:**
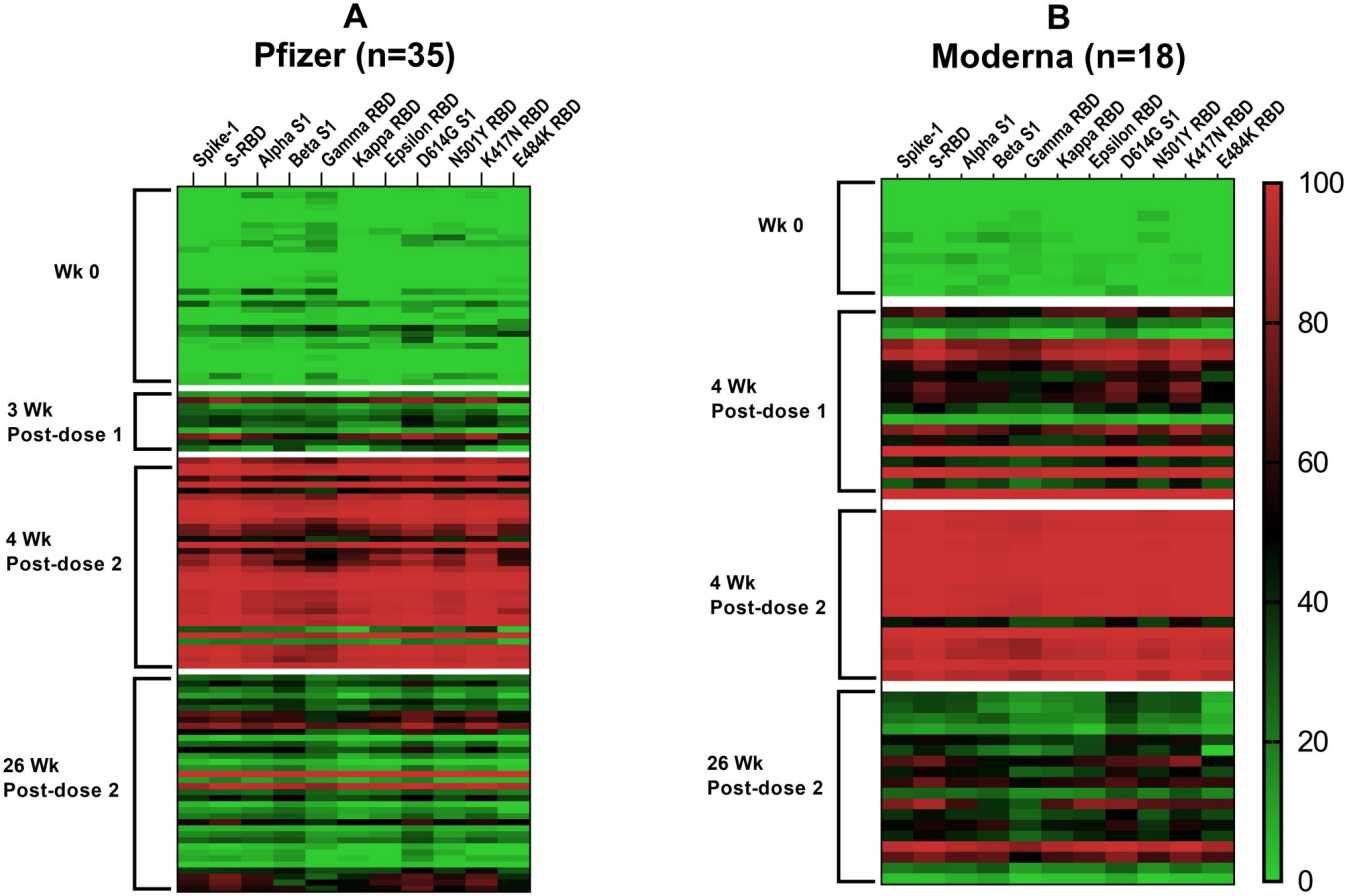
Neutralization capacity of plasma samples collected longitudinally from individuals immunized with Pfizer (A) and Moderna (B) vaccines. Heat maps show the % inhibition data obtained using the sVNT assay for SARS-CoV-2 wildtype (S1 and S-RBD) and nine variants of either S1 or RBD protein subunits. Each row in the heatmap corresponds to one sample and columns correspond to SARS-CoV-2 wild type or variants in the Bio-Plex sVNT assay. The description of samples is as in Fig 2. The color intensity scale represents the % inhibition values ranging from the highest (100; red) to no % inhibition (0; green).

The neutralization capacity of antibodies in the Moderna group (Fig 6B) was about 2-fold higher than the Pfizer group (Fig 6A) at 4 weeks post-first dose for the wildtype SARS-CoV-2 (S1 and S-RBD). A similar trend in the neutralization capacity was noted between the Moderna and Pfizer groups for all the nine SARS-CoV-2 variants tested (1.8 to 3.7-fold). Both Pfizer and Moderna vaccines elicited the highest neutralization capacity at 4 weeks post-second dose (Fig 6A & 6B). However, the neutralization capacity waned at 26 weeks post-second dose vaccination, and the reduction was more in the Pfizer group (63–81%) than in the Moderna group (51–57%) across the tested wild type and variants of SAR-CoV-2.

## Discussion

In this study, we characterized the humoral responses induced by the adenoviral vector-based COVID-19 vaccines in adult macaques and qualitatively compared them to adult humans immunized with Pfizer and Moderna vaccines. We provide evidence for the high efficacy of these vaccines in inducing neutralizing antibodies in the plasma of vaccinated macaques and humans. The vaccine-induced antibody response in the macaques was similar to that of humans. In both species, vaccines elicited low levels of antibodies at 4 weeks post-first dose and strong antibodies at 4 weeks post-second dose. The virus-neutralizing antibody response was highly correlated with the serum antibody titers in both species. In humans, six months after two-dose vaccination, all tested individuals had measurable SARS-CoV-2 antibody levels with a moderate viral-neutralizing capacity. However, 6 months post second-dose vaccine samples of NHP were unavailable to perform the viral-neutralizing capacity in this study. Nonetheless, our results demonstrate the comparative dynamics in antibody response of NHPs after the two-dose vaccination with the Pfizer or Moderna vaccines. Interestingly, a significantly weaker antibody response with an adjusted (lower) dose of Pfizer vaccines in rhesus monkeys, compared to humans, has been reported recently [42].

The NHP models have provided key information on the pathogenesis of SARS-CoV-2 and the immunogenicity of potential vaccines, which is vital to developing therapeutic agents and vaccines for COVID-19 [43–48]. We assessed the S-RBD and N protein adenoviral vector vaccine-induced immune responses over 4 to 6 weeks post-second dose in adult macaques. Both vaccines elicited plasma antibodies at 2 weeks post-first dose in most animals. A strong antibody response was also detected at 2 weeks post-second dose in all vaccinated macaques, and the total IgG (S-RBD) levels strongly correlated with the corresponding neutralizing antibody titers in the plasma.

It has been reported that people living with immunocompromised conditions, such as HIV infection, have worse outcomes after SARS-CoV-2 infection than immunocompetent individuals [49, 50]. This observation has also been confirmed in an immunosuppressed preclinical model of SARS-CoV-2 infection [51]. Hence, vaccination against SARS-CoV-2 in people living with immunocompromised health conditions has become a significant challenge, which requires detailed documentation of the immunogenicity elicited in these individuals by various vaccine platforms. We also studied the humoral immune response to S-RBD adenoviral vector vaccine in SIV-infected adult macaques to determine whether cART treatment impacts the vaccine-induced immune responses. We found that the antibody responses against S-RBD IgG (Total) in SIV-infected macaques under cART were similar to that of non-SIV-infected macaques. However, only two animals were used in this study group and hence the results should be interpreted cautiously.

Our multiplex assay also detects antibodies against the N protein, differentiating between vaccinated and infected people [35]. The study participants had steady-state levels of antibodies against the S proteins of common coronaviruses, 229E, NL63, HKU1, and OC43, consistent with other studies (S4 Table in S1 File) [52]. Furthermore, our findings show that neutralizing and IgG antibodies were highly up-regulated after vaccination and all the individuals who were immunized with the Pfizer or Moderna vaccines developed neutralizing and IgG antibodies against SARS-CoV-2 at 4 weeks post-second dose. Both the wild type and each of the variant SARS-CoV-2 was neutralized almost fully by antibodies generated by vaccination. At 26 weeks post-second dose, SARS-CoV-2 S-RBD IgG (Total) and neutralization capacity dropped to 82% and 73%, respectively, in Pfizer, and 72% and 60%, respectively, in the Moderna group compared to 4 weeks post-second dose. These data are consistent with previous reports of much larger serological studies [6, 53–55]. We also noted that the total IgG antibody levels and neutralization titers were highly correlated in both vaccine groups, consistent with other studies [56–58]. However, the correlation was the least at 4 weeks post-second dose because of the highest antibody levels than the other time points. As all the samples were collected before November 2021, before the spread of the Omicron variant, neutralization capacity against Omicron was not assessed in this study.

Even though both the Pfizer and Moderna vaccines elicited robust immune responses to SARS-CoV-2, several subtle differences emerged between the two groups. The antibody levels were significantly higher in Moderna compared with the Pfizer group at 4 weeks post-second dose, which is consistent with findings from other studies [53, 55]. One of these studies reports that the IgG levels in recipients of the Pfizer vaccine dropped significantly than in Moderna as soon as 21 days post-second dose [53]. Another study reported a significantly higher IgG titer at 6–10 weeks post-second dose among recipients of the Moderna vaccine compared to the Pfizer vaccine [55]. This may be due to the higher dose of the Moderna vaccine (100 μg) compared to Pfizer (30 μg) [2, 3]. In the phase 1 trial, Moderna compared 25μg, 100μg, and 250μg doses and found that the 100μg dose elicits high neutralization responses and Th1-skewed CD4 T cell responses, coupled with less adverse reaction than that of the higher dose [59]. Pfizer evaluated 10μg, 20μg, and 30μg dose levels of the vaccine and found that 30μg dose has a favorable balance of reactogenicity and immunogenicity [60]. Although no specific IgG value correlates with immune protection, observed differences in antibody levels suggest that the Moderna vaccine could promote more durable humoral immunity than the Pfizer vaccine. Reports indicate that breakthrough COVID-19 cases are fewer in individuals immunized with the Moderna vaccine than with the Pfizer vaccine [61, 62].

We observed low levels of IgM antibodies in individuals immunized with Pfizer and Moderna vaccines. This finding is consistent with SARS-CoV-2 infected patients, where the IgM antibodies either appeared after the IgG or coincided with it [35]. Most of the detectable total IgG in both the Pfizer and Moderna vaccinated groups seems to be contributed by IgG1 and, to some extent, by IgG3, similar to natural SARS-CoV-2 infection [35, 63]. These results indicated that the class-switch antibody responses are similar after vaccination with the Pfizer or Moderna vaccines and after a natural infection. IgG3 antibody levels were higher in Moderna compared to Pfizer. Although IgG2 levels were not reported to be elevated in COVID-19 patients [35], we found significantly higher IgG 2 levels in the plasma of the Moderna group compared to the Pfizer group at 4 weeks post-second dose vaccination [64].

Based on our observations, it appears that among the various IgG subtypes, IgG 1 and 3 contribute to neutralization capacity to a lesser extent; at 26 weeks post-second dose, even though the neutralization capacity of the total IgG was strong, the neutralization capacity of IgG1 and IgG3 had dropped. Thus, we predict that IgG1 and IgG3 may play a key role in host protection against the progression of SARS-CoV-2 infection, while IgG4 and IgM seem to have little or no role in vaccine-induced protective efficacy. However, validated immunological reagents to test these antibody sub-types were not commercially available for macaques.

The strong correlation between antibody levels and neutralizing activity in our study supports the use of easy-to-measure antibody levels as the primary metric for defining a correlate of protection in humans and macaques for the mRNA and adenoviral vector-based vaccines. It has been reported that the S-specific antibody responses elicited by the Moderna vaccine correlated with upper and lower airway control of SARS-CoV-2 replication in macaques after the challenge [46]. A recent study reported that the Moderna vaccine-elicited antibody responses restrict viral replication and lung inflammation following SARS-CoV-2 exposure in rhesus macaques [25]. These studies suggest that continuous monitoring of the antibody levels might be a good indicator to guide personalized needs for further booster vaccines to maintain durable and sustained adaptive immunity.

Our study also has certain limitations. Although accumulating evidence suggests that IgG response correlates with protection [65], cellular immunity has also been suggested to play an important role in protecting against SARS-CoV-2 [66]. Thus, a limitation of this study is the lack of data on the parameters of cellular immunity. Another weakness is the limited sample size, and the current study did not evaluate the immune responses elicited by the adenoviral vector (J&J) and recombinant vaccine platforms (Novavax) in humans, which is due to the unavailability of samples from individuals immunized with these vaccines.

## Conclusion and future directions

In summary, this study highlights the role of humoral immune response elicited during immunization of the most commonly used COVID vaccines in a non-human primate model and humans. We provide compelling evidence that key antibody pathways are indeed modulated to various extents during COVID-19 vaccination, which is further impacted by the time post-vaccination. Our data suggest that vaccination could elicit similar antibody class-switching as observed during natural SARS-CoV-2 infection, although the contribution of such a process to the long-term immunity against infection and the duration of antibody responses need additional studies.

## Supporting information

S1 Fig Determination of optimal sample dilution for sVNT assay. % inhibition data obtained using the sVNT assay for SARS-CoV-2 S1 protein plotted against the dilution series for patient samples. Four COVID-19 patient plasma samples are shown each at three different time points post-symptoms (PS).
(TIF)

S2 Fig Correlation between whole virus neutralization titers (WVNT) ID80 with % inhibition of neutralizing antibodies against SARS-CoV-2 S1 in sVNT assay in human COVID-19 patients. The description of samples is as in S1 Fig R^2^ values are shown as an indicator of correlation between the WVNT and sVNT.
(TIF)

S3 Fig Correlations between neutralizing antibody titers and total IgG in adult macaques immunized with adenoviral vector vaccines expressing S-RBD (A) non-SIV infected (n = 7) and (B) SIV-infected animals (n = 2). % inhibition data obtained using the sVNT assay for SARS-CoV-2 S-1 protein plotted against SARS-CoV-2 S-RBD IgG (MFI values) determined by multiplex assays for the same samples. Longitudinal data for each animal is shown. R^2^ values are shown as an indicator of the correlation between the neutralization potency and IgG.
(TIF)

S4 Fig Antibodies (IgG) against SARS-CoV-2 N in humans immunized with mRNA vaccines. A) Pfizer, and B) Moderna (n = 18). The description of the multiplex antibody assay is as in Fig 1. The description of sample time points is as in Fig 2. The dotted line indicates the assay cut-off level calculated using healthy controls (n = 101).
(TIF)

S1 File (XLSX)
