# Supplementary figures and images for: Dynamics of temporal immune responses in nonhuman primates and humans immunized with COVID-19 vaccines
Source: PLoS One. 2023 Oct 19;18(10):e0287377. doi: 10.1371/journal.pone.0287377 (PMC10586671; doi:10.1371/journal.pone.0287377)

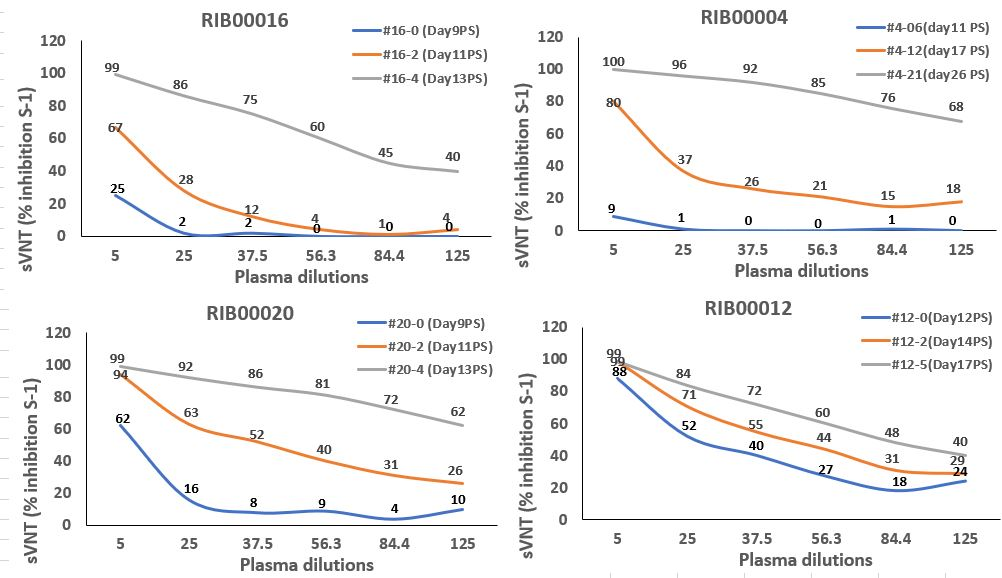

Supplement: S1 Fig — % inhibition data obtained using the sVNT assay for SARS-CoV-2 S1 protein plotted against the dilution series for patient samples. Four COVID-19 patient plasma samples are shown each at three different time points post-symptoms (PS). (TIF) [file pone.0287377.s001.tif]

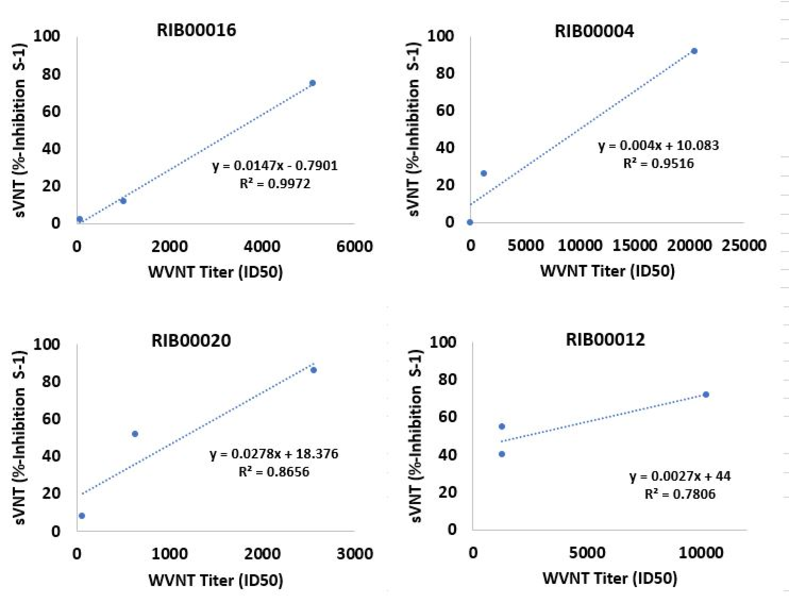

Supplement: S2 Fig — The description of samples is as in S1 Fig R2 values are shown as an indicator of correlation between the WVNT and sVNT. (TIF) [file pone.0287377.s002.tif]

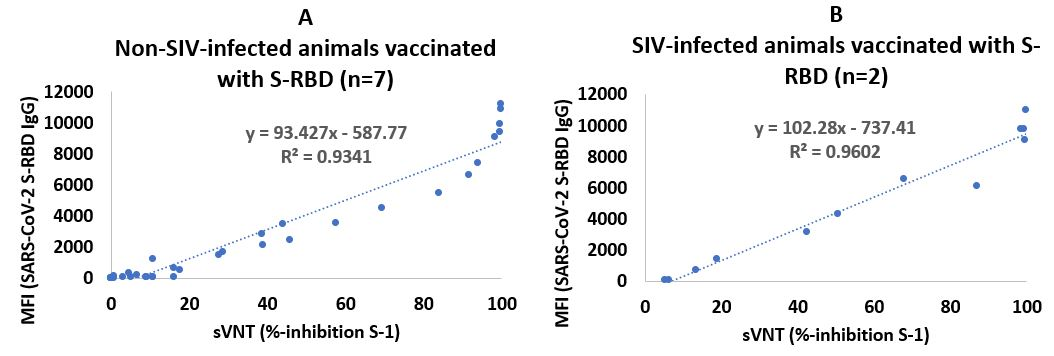

Supplement: S3 Fig — % inhibition data obtained using the sVNT assay for SARS-CoV-2 S-1 protein plotted against SARS-CoV-2 S-RBD IgG (MFI values) determined by multiplex assays for the same samples. Longitudinal data for each animal is shown. R2 values are shown as an indicator of the correlation between the neutralization potency and IgG. (TIF) [file pone.0287377.s003.tif]

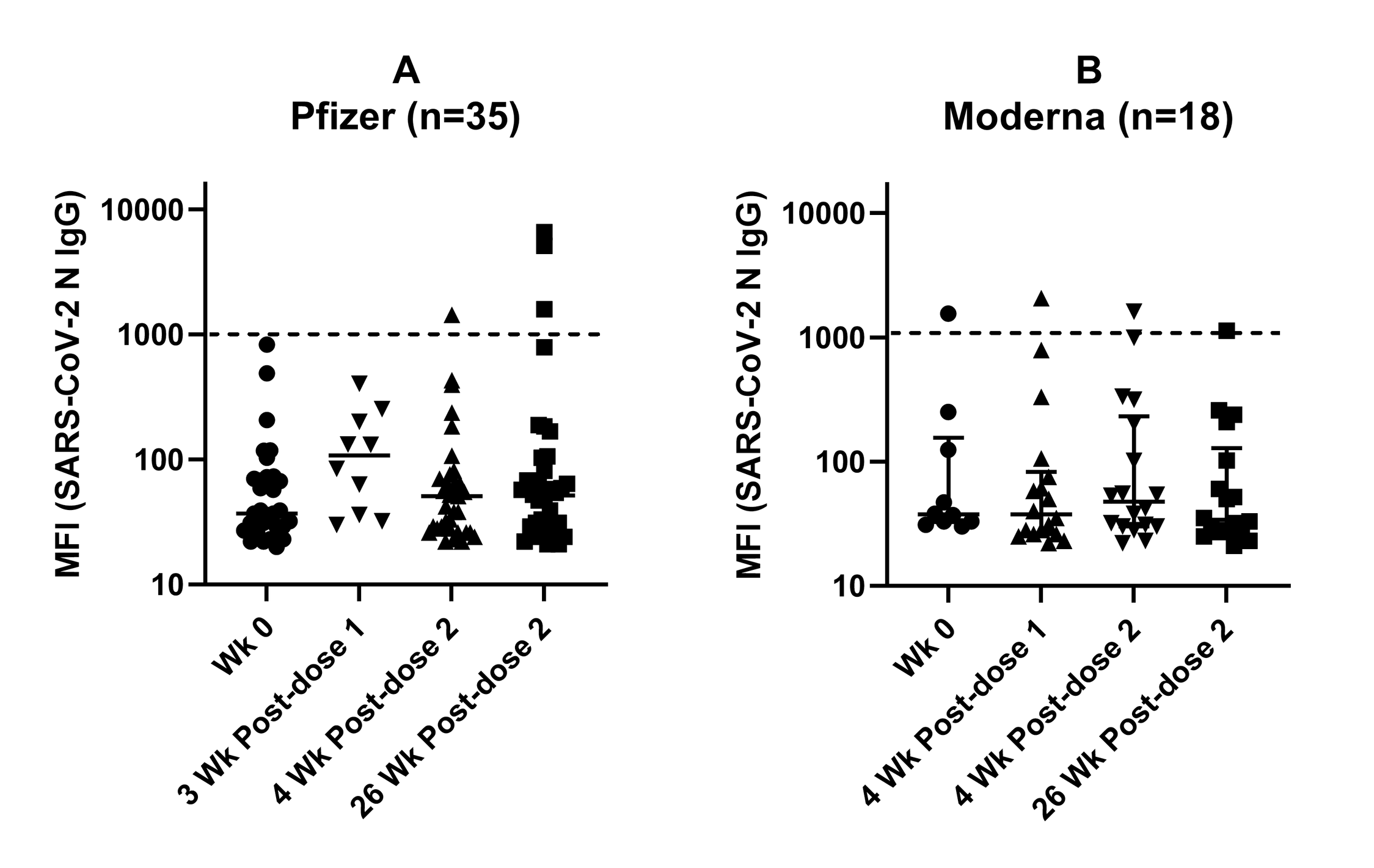

Supplement: S4 Fig — A) Pfizer, and B) Moderna (n = 18). The description of the multiplex antibody assay is as in Fig 1. The description of sample time points is as in Fig 2. The dotted line indicates the assay cut-off level calculated using healthy controls (n = 101). (TIF) [file pone.0287377.s004.tif]
